# Therapeutic Opportunities of Interleukin-33 in the Central Nervous System

**DOI:** 10.3389/fimmu.2021.654626

**Published:** 2021-05-17

**Authors:** Yun Sun, Yankai Wen, Luxi Wang, Liang Wen, Wendong You, Shuang Wei, Lin Mao, Hao Wang, Zuobing Chen, Xiaofeng Yang

**Affiliations:** ^1^ Department of Rehabilitation Medicine, The First Affiliated Hospital, Zhejiang University, Hangzhou, China; ^2^ Department of Anesthesiology, McGovern Medical School, University of Texas Health Science Center at Houston, Houston, TX, United States; ^3^ Department of Neurology, The First Affiliated Hospital of Wenzhou Medical University, Wenzhou, China; ^4^ Department of Neurosurgery, The First Affiliated Hospital, Zhejiang University, Hangzhou, China

**Keywords:** interleukin-33, ST2, multiple sclerosis, ischemic stroke, trauma, hemorrhage, Alzheimer’s disease, anti-inflammatory macrophages

## Abstract

Interleukin-33 (IL-33), a member of the IL-1 cytokine family, is involved in various diseases. IL-33 exerts its effects via its heterodimeric receptor complex, which comprises suppression of tumorigenicity 2 (ST2) and the IL-1 receptor accessory protein (IL-1RAP). Increasing evidence has demonstrated that IL-33/ST2 signaling plays diverse but crucial roles in the homeostasis of the central nervous system (CNS) and the pathogenesis of CNS diseases, including neurodegenerative diseases, cerebrovascular diseases, infection, trauma, and ischemic stroke. In the current review, we focus on the functional roles and cellular signaling mechanisms of IL-33 in the CNS and evaluate the potential for diagnostic and therapeutic applications.

## Introduction

**Figure 1:**
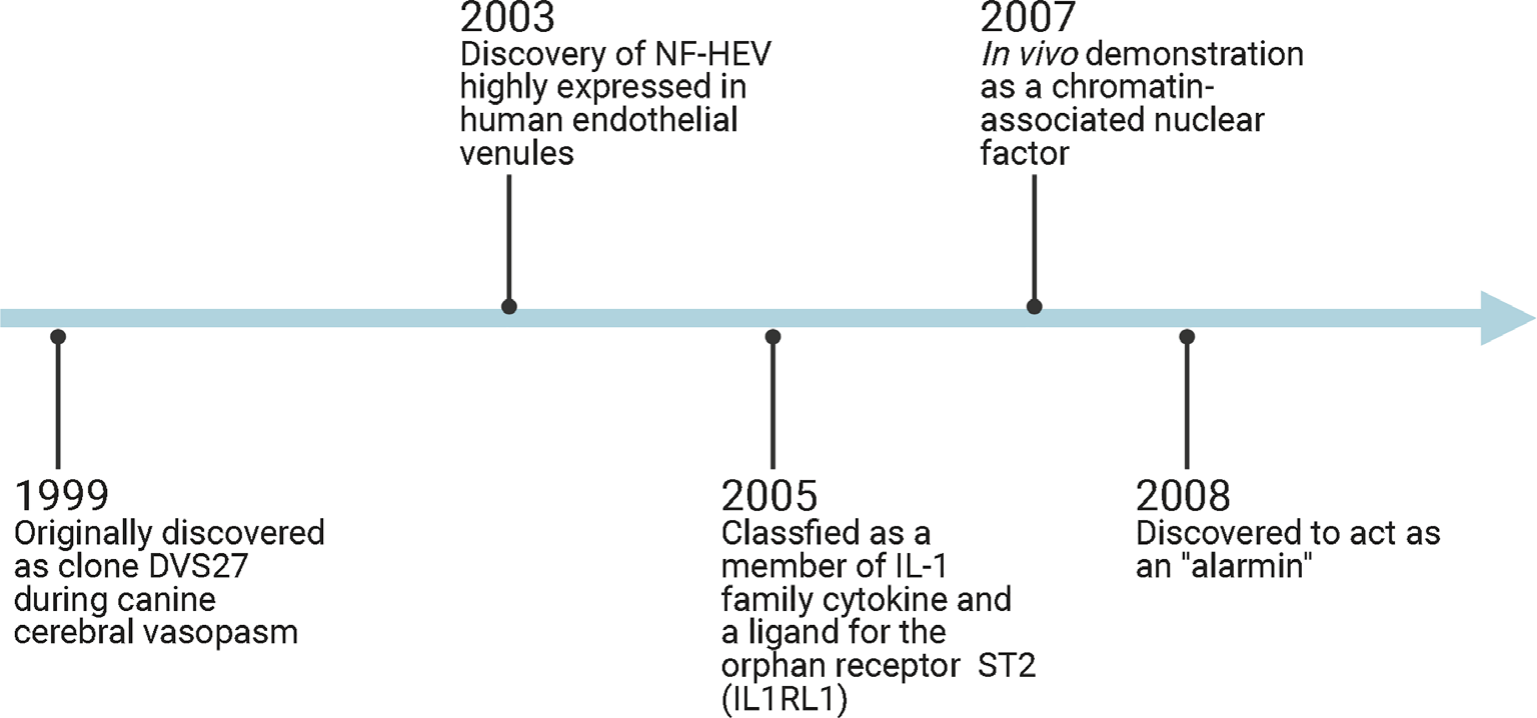
Timeline diagram demonstrates IL-33-related discoveries. HF-HEV: nuclear factor from high endothelial venules; IL1RL1: interleukin 1 receptor-like 1; ST2: suppression of tumorigenicity 2.

**Figure 2:**
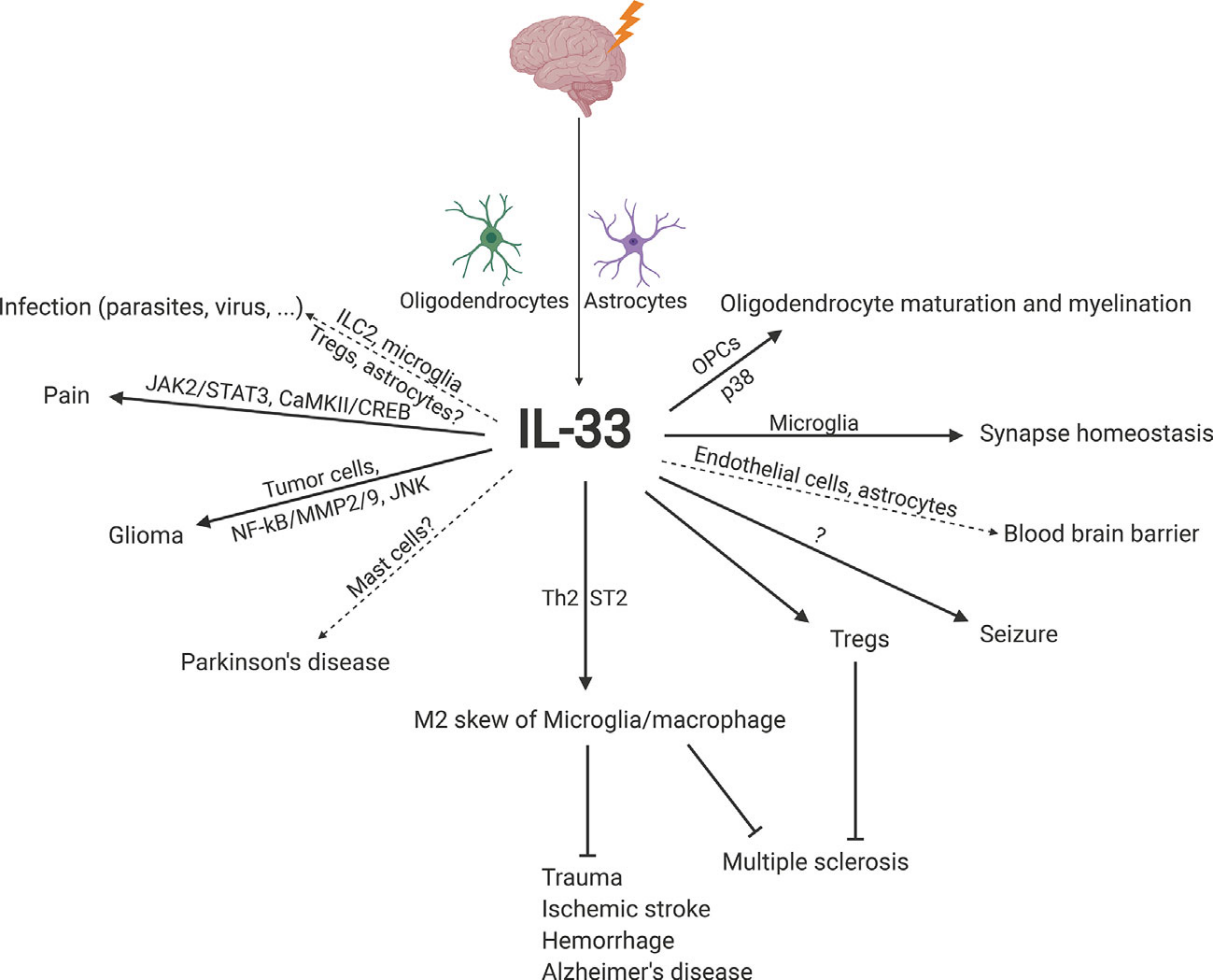
Schematic overview of the roles and underlying signaling mechanisms of IL-33 in CNS homeostasis and disease. CaMKII, calcium-calmodulin-dependent kinase II; CREB, cyclic adenosine monophosphate response element-binding protein; IL-33, interleukin-33; ILC2, group 2 innate lymphoid cell; JAK2, Janus kinase 2; JNK, c-Jun N-terminal kinase; MMP2/9, matrix metalloprotease 2/9; NF-κB, nuclear factor-κB; OPC, oligodendrocyte precursor cell; ST2, suppression of tumorigenicity 2; STAT3, signal transducer and activator of transcription 3; Th2, T helper type 2; Treg, regulatory T cell.

Interleukin-33 (IL-33) was originally discovered in 1999 as clone DVS27 in vasospastic cerebral arteries in a canine model of subarachnoid hemorrhage (SAH) (1) (**Figure 1**). Then, in 2003, IL-33 was identified as nuclear factor from high endothelial venules (NF-HEV), a nuclear factor preferentially expressed in human high endothelial venules (2). In 2005, IL-33 was classified as a member of the IL-1 cytokine family and named IL-1F11 (3). Remarkably, IL-33 is continuously expressed in healthy brains and spinal cords (4), which have higher IL-33 mRNA expression than any other tissue tested (3). In recent years, IL-33 has been found to be involved in various disorders of the central nervous system (CNS), including multiple sclerosis (MS) (5, 6), infection (7, 8), ischemic stroke (9, 10), traumatic brain injury (TBI) (11, 12), spinal cord injury (SCI) (4, 13), brain tumorigenesis (14, 15), and mental disorders (16) (**Figure 2**). In the current review, we will focus on the functional roles and cellular signaling mechanisms of IL-33 in the CNS and evaluate the potential for diagnostic and therapeutic applications.

## IL-33 and Its Receptor

Human IL-33 is located on chromosome 9p24.1 and encodes 270 amino acids, while its mouse counterpart is located on the syntenic chromosome 19qC1 region and encodes 266 amino acids. At the amino acid level, human and mouse IL-33 are 55% identical (3). IL-33 can be confined in the nucleus as a result of binding heterochromatin with an evolutionarily conserved homeodomain-like helix-turn-helix motif within its N-terminal region (17). However, under pathological conditions, IL-33 is released by stressed or damaged cells as an alarmin (18–20). IL-33 is released in either a full-length or cleaved form. Unlike IL-1β, IL-33 is not cleaved by caspase-1 (21). Instead, processing by caspase-1, caspase-3, and caspase-7 actually leads to IL-33 inactivation (22, 23). Other proteins, such as neutrophil elastase, cathepsin G (24), chymase and tryptase (25, 26), cleave IL-33, and the resulting cleaved IL-33 has much higher biological activity than full-length IL-33.

Extracellular IL-33 exerts its effects by binding to the orphan receptor suppression of tumorigenicity 2 (ST2, also known as IL1RL1, DER4, T1 and FIT-1) (3). Upon binding IL-33, ST2 on the cell membranes forms a heterodimer with IL-1 receptor accessory protein (IL-1RAP) (27, 28), which leads to the dimerization of the Toll/interleukin-1 receptor (TIR), the subsequent recruitment of myeloid differentiation primary response protein 88 (MyD88) and the activation of IL-1R-associated kinase, and ultimately activate the mitogen-activated protein kinase (MAPK) and nuclear factor-κB (NF-κB) signaling pathways (3, 29).

Notably, four isoforms of ST2 exist: transmembrane ST2L, soluble ST2 (sST2), ST2V and ST2LV. ST2L and sST2 come from a dual promoter system to drive differential mRNA expression (30). Transmembrane ST2L contains transmembrane and cytoplasmic domains that are absent in sST2 (31). sST2 has been reported to be a decoy receptor that competes with ST2L for IL-33 binding, thus inhibiting the IL-33 signaling pathway (32, 33). ST2V and ST2LV are two splice variants of ST2. ST2V gains a hydrophobic tail instead of losing the third immunoglobulin-like domain in the C-terminal portion of ST2L (34), while ST2LV is produced from deletion of the transmembrane domain of ST2L (35). To date, there have been no reports in the literature of the interaction of IL-33 with ST2V or ST2LV.

In addition to sST2, other factors have been reported to inhibit IL-33/ST2 signaling by regulating ST2. For example, single immunoglobulin domain IL-1R-related molecule (SIGIRR, also known as TIR8) forms a complex with ST2 upon IL-33 stimulation and subsequently inhibits IL-33-mediated signaling (36, 37). Another negative regulator is F-box and leucine-rich repeat protein 19 (FBXL19), which mediates the ubiquitination and degradation of ST2 (38).

## IL-33 in CNS Development and Homeostasis

IL-33 expression was first detected in the mouse CNS during late embryogenesis, and its expression increased from postnatal day 2 (P2) to P9 then declined and became absent after P23. During this period, astrocytes and oligodendrocyte precursor cells (OPCs) rather than neurons are responsible for IL-33 expression (39, 40). IL-33 induces the expression of myelin basic protein and the transcription of myelin genes via p38 MAPK activation in OPCs *in vitro* (41). Conversely, IL-33 deficiency disrupts OPC differentiation into oligodendrocytes and interferes with myelin compaction *in vitro* and *in vivo* (42), indicating a potential role of IL-33 in oligodendrocyte maturation and myelination during CNS development. During the early period of postnatal synapse maturation, IL-33 expression increases in astrocytes but only in a subpopulation of spinal cord and thalamic astrocytes in gray matter, where most synapses are located. This IL-33 induction is developmentally crucial for neural circuit function and ST2-expressing microglial synapse engulfment in the spinal cord and thalamus. IL-33-deficient mice have deficits in the acoustic startle response, which is a sensorimotor reflex mediated by motor neurons in the brainstem and spinal cord. Furthermore, in IL-33-deficient mice, spontaneous and evoked oscillatory activity increases in an intrathalamic circuit between the ventrobasal nucleus and the reticular nucleus of the thalamus (40), which implies that IL-33 is required to maintain synapse homeostasis during CNS development.

IL-33 is constitutively expressed in the corpus callosum, hippocampus, thalamus, and cerebellum (granular layer and white matter) in adulthood, which was discovered using IL-33-LacZ gene trap reporter adult mice (43). It is predominantly expressed in astrocytes in both the brain and spinal cord in mice. IL-33 is also expressed in oligodendrocytes, microglia, and neurons, but at a lower level (16). ST2 expression is found in neurons and glial cells in the brain and spinal cord in rodents. Notably, the expression of ST2 on cerebral endothelial cells and astrocytes close to the endothelial layer of the cortex implies a role of IL-33/ST2 signaling in maintaining the function of the blood brain barrier (BBB) (16, 44). Despite the fact that IL-33 protein is always localized in the nucleus of cells as an alarmin (43), whether the nuclear IL-33 impacts CNS homeostasis is unclear.

## IL-33 in Multiple Sclerosis

Multiple sclerosis (MS) is an autoimmune disease characterized by demyelination and neurodegeneration in the CNS. IL-33 is elevated in plasma, normal-appearing white matter, and lesions in MS patients compared to normal controls (5, 45). In experimental autoimmune encephalomyelitis (EAE) mice, a widely used mouse model of MS, the expression levels of IL-33 and ST2 in the spinal cord are elevated compared to those in naïve mice (46, 47).

The role of IL-33/ST2 signaling in MS has been intensively investigated in mice. Although one group demonstrated that blockade of IL-33 protected mice against EAE (46), most literature suggests that IL-33 is a protective factor. Exogenous IL-33 can significantly suppress EAE in mice (48–50). Conversely, either IL-33 antibody or genetic ablation exacerbates EAE development in mice (47, 51). In addition, ST2-deficient mice develop aggravated EAE that cannot be restored by exogenous IL-33 injection (48, 52). Several mechanisms are involved in IL-33-attenuated EAE. First, IL-33 reduces the frequency of IL-17A^+^ and interferon gamma^+^ (IFNγ^+^) cells in both draining lymph nodes (DLNs) and spleens (48, 51, 52). This is probably due to the attenuated inflammatory phenotype of antigen-presenting cells (APCs) upon IL-33/ST2 activation. Transplantation of dendritic cells (DCs) from DLNs and spleens of ST2-deficient MOG_35-55_-immunized EAE mice to wild-type counterparts exacerbates EAE development (52). Second, IL-33 protects EAE mice via regulatory T cells (Tregs), which are ST2 positive (53). IL-33 significantly increases the frequency of Tregs in the spinal cords of EAE mice (48). Consistent with these results, Tregs are enriched within MS lesions but not in remyelinating lesions in patients and produce IL-10, which facilitates the resolution of MS (54). Third, IL-33 polarizes macrophages in DLNs and spleens toward the anti-inflammatory M2 phenotype. Transplantation of macrophages from lymph nodes (LNs) and spleens of IL-33-treated mice to immunized mice attenuates EAE development (48). Last, other cell types educated by IL-33 exert protective roles. For example, IL-33-treated eosinophil transplantation confers protection against EAE in mice (50). ST2-expressing mast cells, group 2 innate lymphoid cells (ILC2s) and basophils are also implied to produce anti-inflammatory IL-4 and/or IL-13 to protect against EAE in mice (49).

Furthermore, experiments using rat *in vitro* myelinating coculture have suggested that IL-33 inhibits axon myelination during MS pathogenesis (45). However, IL-33 has also been reported by another group to promote myelin repair (41). Therefore, the direct effect of IL-33 on myelination and the underlying mechanisms need to be further investigated. Taken together, these studies show that IL-33 has a protective effect in a mouse model of EAE and might be a promising therapeutic target for MS patients.

## IL-33 in Trauma

IL-33 has been reported to be increased in microdialysate samples of patients with TBI (11). Serum sST2 levels are also elevated in TBI patients, and the increased concentrations are positively related to inflammation, severity and prognosis (55), which indicates that sST2 is a potential prognostic biomarker for TBI. In mice, IL-33 is released immediately from damaged oligodendrocytes and astrocytes in injured CNS tissue during TBI induced by controlled cortical impact (CCI) and experimental SCI (4, 11, 13, 56).

After CCI or SCI, ST2- or IL-33-deficient mice exhibit attenuated induction of chemokines in local astrocytes, such as C-C motif chemokine ligand 2 (CCL2), which subsequently impairs inflammatory monocyte infiltration (4, 11). Consistent with these results, treatment with recombinant IL-33 alleviates secondary damage by significantly decreasing tissue loss, demyelination and astrogliosis in the contused mouse spinal cord, which contributes to improved functional recovery (13). IL-33 prevents TBI-induced inflammation and apoptosis in mice (56). In addition, IL-33 augments the skew of macrophages toward the M2 phenotype (4, 13), which is beneficial after CNS injury. However, the underlying mechanism by which IL-33/ST2 signaling drives the anti-inflammatory response in CNS trauma needs to be further investigated.

## IL-33 in Ischemic Stroke

The concentrations of both IL-33 and sST2 increase in circulating blood in patients with ischemic stroke (9, 10, 57, 58), and both are negatively correlated with patient outcome and can serve as independent diagnostic and predictive prognostic markers in ischemic stroke patients (10, 58, 59). Besides, two ongoing clinical trials in United States (NCT03297827) and Poland (NCT03948802) are evaluating the utility of IL-33 as a biomarker of acute stroke. And another active clinical trial in Croatia (NCT04607031) is determining the prognostic accuracy of sST2 dynamics in ischemia stroke outcomes. The results from these clinical trials will provide further evidence to determine whether IL-33 and sST2 are the reliable biomarkers for stroke patients.

In the mouse model of ischemic stroke induced by middle cerebral artery occlusion (MCAO), IL-33 mRNA and protein expression are obviously upregulated in lesions, and mature oligodendrocytes and astrocytes are responsible for this upregulation (9, 60–62).

ST2 or IL-33 deficiency exacerbates ischemic brain injury after MCAO in mice (60, 61). Conversely, exogenous IL-33 treatment protects mice against experimental ischemic stroke (9, 63–65) and neonatal hypoxic ischemic brain injury (62). Mechanistically, IL-33 does not directly act on neurons. Instead, similar to trauma, IL-33 reduces astrocyte activation and drives the type 2 response (9). Two pathways might be involved in the IL-33-driven skew of microglia/macrophages toward the M2 phenotype after MCAO. First, it has been reported that IL-33 increases peri-ischemic IL-4 secretion from T cells in the brain, contributing to the skew toward M2 macrophages. Moreover, the IL-4 antibody treatment abrogates IL-33-mediated protection in mice after MCAO (9). Second, IL-33 directly potentiates M2 polarization of microglia/macrophages via an unknown signaling pathway after MCAO (57, 60). Another mechanism involved in IL-33-mediated protection in ischemic stroke is promotion of the T helper type 2 (Th2) response and suppression of Th1 and Th17 responses (63, 65).

Surprisingly, blockade of CD25 on Tregs fails to abrogate IL-33-mediated protection in mice after MCAO (9), even though ST2^+^ Tregs are observed to increase in ischemic brain and spleen in MCAO mice upon IL-33 treatment (64–67) and IL-33/ST2 signaling has been suggested to play an important role in the expansion and function of brain Tregs (68); this result indicates that the expanded Tregs in MCAO mice are only bystanders. In summary, IL-33 treatment might be a promising therapeutic method for ischemic stroke patients.

## IL-33 in Hemorrhage

As previously described, IL-33 was initially identified as one of the differentially expressed genes in vasospastic cerebral arteries after SAH in dogs (1). It was then reported that IL-33 protein and mRNA levels increase in the brain cortex in a rat model of SAH, and IL-33 is mainly localized in neurons (69). However, in the rodent model of intracerebral hemorrhage (ICH), IL-33 mainly localizes in astrocytes and microglia rather than in neurons in the brain around the hematoma of the ipsilateral hemisphere. Furthermore, exogenous IL-33 exerts a neuroprotective effect against ICH via selective microglial M2 polarization and subsequent inflammatory suppression (70, 71).

## IL-33 in Infection

The cerebral complications of malaria, which is caused by *Plasmodium falciparum* infection, are associated with long-term neurological sequelae in survivors. IL-33 expression in oligodendrocytes and astrocytes is increased in the mouse brain during *Plasmodium berghei* ANKA (PbA) infection-induced experimental cerebral malaria (ECM) as well as during other parasitic infections, including *Toxoplasma gondii* and *Angiostrongylus cantonensis* (8, 72–75). ST2 deficiency disrupts ECM infection, prevents recognition impairment and improves survival in mice with PbA infection, and these effects might be attributed to the suppression of IL-1β production by ST2-deficient microglia and the impaired sequestration and activation of pathogenic T cells (8, 72). Interestingly, two other groups reported other scenarios. One group used IL-33-deficient mice to demonstrate that IL-33 was not critical for ECM development (76). The other group elucidated that exogenous IL-33 protected mice from PbA infection-induced ECM by orchestrating a protective immune response via ILC2s, M2 macrophages and Tregs (77). These results are supported in *Toxoplasma gondii*-infected mice. Deletion of ST2 increases susceptibility to *Toxoplasma gondii* infection in mice due to the increased parasite burden in the brain (74, 75). Moreover, astrocytes without ST2 fail to recruit and/or maintain adequate antiparasitic IFNγ-expressing T cells and inducible nitric oxide synthase (iNOS)-expressing monocytes/macrophages in the CNS to control parasites (75).

Bacterial infection may induce endotoxemia, leading to neuroinflammation. Intracerebroventricular endotoxin (also known as lipopolysaccharide, LPS) induces IL-33 production by glia in the brain (20, 44). IL-33-deficient mice exhibit attenuated neuroinflammation upon LPS challenge. Microglia are the target cells that are stimulated by IL-33 to produce proinflammatory cytokines during LPS stimulation (44).

Furthermore, IL-33/ST2 signaling plays a role in virus-induced CNS diseases. In the newborn brain with Zika virus-induced microcephaly, IL-33 is significantly upregulated compared to that in newborn brains with microcephaly without virus infection (78, 79). IL-33 has been found to positively correlate with IL-1β expression (79, 80), implying that IL-33 might play a role in the proinflammatory response in virus infection. In addition, an *in vitro* study demonstrated that HIV infection induces IL-33 release from neurons and ST2 upregulation in astrocytes. A higher IL-33 concentration is associated with decreased synaptic plasticity due to enhanced neuroinflammation (81), indicating a deleterious role of IL-33 in neuropathogenesis in HIV infection. However, in an experimental mouse model of encephalitis induced by Rocio virus infection, ST2 knockout mice showed increased susceptibility to infection and an increased mortality rate, possibly attributable to increased iNOS production through local IFNγ modulation (7). Taken together, these data suggest that more investigations are warranted to determine the role of IL-33/ST2 signaling in parasitic/viral infection.

## IL-33 in Tumors

Glioma is the most frequent intracranial tumor in adult humans. It has been reported that IL-33 and ST2 expression in glioma tissues is higher than that in normal brain tissues, and their expression is positively correlated with glioma grade (15, 82). Moreover, higher IL-33 expression is associated with poor overall survival (OS) and recurrence-free survival (RFS) in patients with gliomas (14, 15, 83), indicating that IL-33 might be an independent prognostic marker for glioma.

IL-33 is highly expressed in tumor cells during glioma development. It is believed that glioma cells are also ST2 positive. Knockdown of IL-33 or ST2 in glioma cell lines suppresses proliferation, migration and invasion *in vitro* and reduces tumor formation *in vivo* in both rodent models of intracerebral glioma cell implantation and subcutaneous xenograft (82–84). Mechanistically, IL-33/ST2 activates NF-κB signaling to induce matrix metalloprotease 2/9 (MMP2/9) to enhance cell migration and invasion (82) and promotes c-Jun N-terminal kinase (JNK) signaling to induce the expression of key transcription factors that control the process of epithelial-to-mesenchymal transition (EMT) and stemness (83). Notably, IL-33 prevents temozolomide (TMZ)-induced brain tumor apoptosis, and blocking IL-33/ST2 signaling can increase the sensitivity of tumors to TMZ (83). On the other hand, a recent study demonstrated that glioma-derived IL-33 correlated with increased tumor-associated macrophages/microglia in human specimens and in mice with intracerebral xenografts. The group reported that ST2 expression was minimal on glioma cells and that nuclear IL-33 mediated the release of inflammatory cytokines from glioma cells and was required for the recruitment of M2 protumorigenic macrophages (85).

An anticancer role of ST2 has been proposed. ST2 binds to tumor cell apoptosis factor (TCApF), a peptide naturally expressed in the frontal lobe of the brain, to activate caspase-3-, caspase-8-, and caspase-9-mediated apoptosis (86). However, whether IL-33 competes with TCApF for ST2 binding in glioma is unclear. Nevertheless, IL-33 might be a promising therapeutic target for glioma.

## IL-33 in Alzheimer’s Disease and Parkinson’s Disease

Although IL-33-positive cells (astrocytes and microglia) are significantly increased in Alzheimer’s disease (AD) brains compared to non-AD brains (87), IL-33 mRNA expression in the brain (88) and circulating IL-33 levels (89) are lower in AD patients than in healthy controls. The decreased circulating IL-33 levels in AD might be attributed to the increased decoy receptor sST2 in the blood of AD patients (89). Three single nucleotide polymorphisms (SNPs), rs1157505, rs11792633 and rs7044343, within IL-33 have been reported to be associated with AD risk in a large prospective study in a Caucasian population. These polymorphisms are associated with less cerebral amyloid angiopathy (CAA) in the brain (88), which correlates closely with AD pathology. These SNPs were further evaluated in two independent cohorts in the Han Chinese population. It was reported that the IL-33 rs11792633 polymorphism was significantly associated with a reduced risk of late onset AD (LOAD) in patients and that the T allele was a protective factor for LOAD (90, 91).

IL-33 has been suggested to prevent AD development in *in vitro* and *in vivo* animal studies. IL-33 overexpression in *in vitro* cellular models induces a specific decrease in the secretion of amyloid β_40_ (Aβ_40_) peptides, which are the main component of CAA (88). In the APPswe, PSEN1dE9 (APP/PS1) double transgenic mouse, an AD mouse model, IL-33 administration reduces soluble Aβ levels and amyloid plaque deposition by enhancing microglial recruitment, Aβ phagocytic activity and anti-inflammatory responses via ST2/MAPK signaling, ultimately contributing to the amelioration of AD development (92). Strikingly, compared to their wild-type counterparts, IL-33-deficient aged mice (aged 65-80 weeks) develop significant abnormal tau accumulation, which is a biomarker for AD (93), and late-onset neurodegeneration in the cerebral cortex and hippocampus accompanied by impaired cognition/memory. IL-33 deficiency induces impaired repair of DNA double-strand breaks and defective autophagic clearance of cellular waste in neurons (94). IL-33 might also contribute to repressing aging-associated neuroinflammation and cognitive decline via ILC2s. The ILC2s are ST2 positive and functionally quiescent at homeostasis but can be activated by IL-33 to produce a vast range of type 2 cytokines to combat aging-associated neurodegenerative disorders (95).

Increased IL-33-expressing astrocytes are detected in the midbrain and striatum of Parkinson’s disease (PD) brains compared with age- and sex-matched control brains (96). *In vitro* studies have shown that 1-methyl-4-phenylpyridinium (MPP^+^), a metabolite of the parkinsonian neurotoxin 1-methyl-4-phenyl-1,2,3,6-tetrahydropyridine (MPTP), induces IL-33 release from astrocytes (97). Mast cells, a population of IL-33-targeting cells whose activation is detected in PD brains, might contribute to neuroinflammation during PD development (96, 97). Together, IL-33 administration could be a potential therapy for AD. However, the role of IL-33 in PD remains elusive.

## IL-33 in Pain

The role of IL-33/ST2 signaling in the pain response has been well characterized in different animal models. In various rodent models, IL-33 expression is increased in neurons, oligodendrocytes and/or astrocytes in the spinal cord (98–101). Either blocking ST2 genetically, intrathecal administration of an ST2-neutralizing antibody or IL-33 knockdown attenuates model-induced mechanical hyperalgesia and heat/cold allodynia (98–102). In the spared nerve injury (SNI) neuropathic pain model, IL-33 activates the astroglial Janus kinase 2 (JAK2)/signal transducer and activator of transcription 3 (STAT3) cascade and the neuronal calcium-calmodulin-dependent kinase II (CaMKII)/cyclic adenosine monophosphate response element-binding protein (CREB) cascade to contribute to nociceptive behaviors (98). On the other hand, IL-33-induced hyperalgesia, which is initiated by SNI (99), bone cancer (100), non-compressive lumbar disk herniation (101), or complete Freund’s adjuvant (102), is inflammation dependent, indicating the involvement of microglia.

## IL-33 in Seizure

In SAH patients, plasma sST2 levels are higher among patients with new or worsening epileptiform abnormalities than those of patients without (103), implying that IL-33, which can be decoyed by sST2, is associated with reduced odds of epileptiform abnormalities. In a rat model of recurrent neonatal seizure (RNS), IL-33 administration restores the reduced IL-33 in the cortex induced by RNS, improves RNS-induced behavioral deficits, promotes body weight gain, and ameliorates spatial learning and memory ability by impeding NF-κB-mediated neuroinflammation (104, 105).

## IL-33 in Mental Disorders

Even though both IL-33 and sST2 levels in chronic schizophrenia patient sera are comparable with those in their control counterparts, serum IL-33 is positively correlated with cognitive performance in patients with schizophrenia (106). Furthermore, the IL-33 gene polymorphism (rs11792633) is associated with the development of schizophrenia. The CT and TT variants of rs11792633 are related with significantly reduced risk of schizophrenia (107).

IL-33 has also been implicated in other kinds of mental disorders. In women with a history of recurrent major depressive disorder, circulating IL-33 is higher than that in healthy controls (108). Another study found that IL-33 concentrations were significantly associated with increased odds of perinatal major depressive episodes (109). However, IL-33 levels in blood in children with autism spectrum disorder do not differ from control counterparts (110, 111). In contrast, elevated IL-33 expression in the brain is observed in BTBR *T+tf*/J (BTBR) mice, which exhibit several symptoms of autism, including reduced social interactions, restricted repetitive behaviors and unusual vocalizations (112). Interestingly, IL-33-deficient mice exhibit reduced anxiety-like behaviors and impaired social novelty recognition via unknown signaling (113).

## IL-33 in Amyotrophic Lateral Sclerosis

IL-33 levels in serum is lower in patients with amyotrophic lateral sclerosis (ALS) than those in healthy controls (114). In the transgenic mice of ALS expressing G93A-superoxide dismutase 1 (SOD1-G93A), long-term IL-33 administration delays disease onset in females but not males probably through peripheral Th2 response (115).

## IL-33 in Neuro-Behçet’s Disease

Neuro-Behçet’s disease (NBD) causes CNS complications and is present in 5-30% of patients with Behçet’s disease (BD) (116). IL-33 has been reported to be significantly higher in the cerebrospinal fluid of patients of NBD compared with those of patients with headache attributed to BD and patients with non-inflammatory neurological diseases. And expression of IL-33 mRNA in cerebrospinal fluid cells from patients with NBD is positively correlated with CCL2 and C-X-C motif chemokine ligand 10 (CXCL10) expression (117). Though IL-33 is implied to play a role in CNS inflammation in NBD patients, future investigations are warranted to determine whether and how the IL-33 plays a beneficial or detrimental role in CNS of patients with NBD.

## Conclusion

IL-33 is induced predominantly in oligodendrocytes and astrocytes in the CNS. In addition to promoting brain tumorigenesis, IL-33 promotes M2 macrophage skew and/or Treg expansion and activation to establish an anti-inflammatory microenvironment against diseases, especially MS, AD, trauma, ischemic stroke, and hemorrhage (**Figure 2**), which suggests that IL-33 administration is an appropriate and desired therapeutic treatment against CNS diseases.

Currently, most studies are linking increased IL-33 induced in injured CNS to anti-inflammatory microenvironment in local lesions and/or lymphatic tissues (spleen and LNs). However, IL-33 is expressed in various organs (such as gut, lung, skin…), and plays a critical role in tissue homeostasis, injury and inflammation (118) as well as controlling gut microbiota (119, 120). Since targeting microbiota-gut-brain axis becomes new therapeutic strategy for neurological diseases (121), in the future, studies on the role of IL-33 from gut in microbiota-gut-brain axis might be emerging. Secondly, given that IL-33 is a powerful cytokine in a variety of organs and disease, the best strategy to deliver IL-33 to suppress inflammation in CNS without inducing side effect in other organs needs to be further investigated. Thirdly, current clinical trials using IL-33/ST2 signaling to treat CNS diseases are totally blank. There is still a long way to go from bench to bedside for IL-33/ST2 therapy in CNS diseases.

## Author Contributions

Literature review and manuscript writing: YS, YW, and LWa. Language editing: LWe, WY, SW, LM, and HW. Design of review outline: XY, ZC, and YS. All authors contributed to the article and approved the submitted version.

## Conflict of Interest

The authors declare that the research was conducted in the absence of any commercial or financial relationships that could be construed as a potential conflict of interest.
